# Cross Talk between ARF1 and RhoA Coordinates the Formation of Cytoskeletal Scaffolds during Chlamydia Infection

**DOI:** 10.1128/mBio.02397-21

**Published:** 2021-12-14

**Authors:** Adam Haines, Jordan Wesolowski, Nathan M. Ryan, Tiago Monteiro-Brás, Fabienne Paumet

**Affiliations:** a Department of Immunology and Microbiology, Thomas Jefferson University, Philadelphia, Pennsylvania, USA; b Life and Health Sciences Research Institute (ICVS), School of Medicine, University of Minho, Braga, Portugal; c ICVS/3B’s, PT Government Associate Laboratory, Braga/Guimarães, Portugal; Yale University School of Medicine

**Keywords:** ARF1, *Chlamydia*, GTPases, RhoA, actin, cytoskeleton, microtubules

## Abstract

Chlamydia trachomatis is an obligate intracellular bacterium that has developed sophisticated mechanisms to survive inside its infectious compartment, the inclusion. Notably, *Chlamydia* weaves an extensive network of microtubules (MTs) and actin filaments to enable interactions with host organelles and enhance its stability. Despite the global health and economic burden caused by this sexually transmitted pathogen, little is known about how actin and MT scaffolds are integrated into an increasingly complex virulence system. Previously, we established that the chlamydial effector InaC interacts with ARF1 to stabilize MTs. We now demonstrate that InaC regulates RhoA to control actin scaffolds. InaC relies on cross talk between ARF1 and RhoA to coordinate MTs and actin, where the presence of RhoA downregulates stable MT scaffolds and ARF1 activation inhibits actin scaffolds. Understanding how Chlamydia hijacks complex networks will help elucidate how this clinically significant pathogen parasitizes its host and reveal novel cellular signaling pathways.

## INTRODUCTION

Actin and tubulin are among the most abundant proteins in eukaryotic cells and are involved in essential cellular processes, including organelle positioning and function. Together, they comprise the most critical components in the cytoskeleton: actin filaments and microtubules (MTs). Additional cellular proteins regulate each cytoskeletal element’s structure, dynamics, and function and, consequently, the fundamental biological processes dependent on the cytoskeleton (1). As a result, these regulatory components of the cytoskeleton are significant targets for intracellular bacterial pathogens (2–4).

Chlamydia trachomatis is the most common cause of bacterial sexually transmitted disease. It is also the most frequent cause of infectious blindness, known as trachoma (5). C. trachomatis displays a unique biphasic lifestyle. The infectious form, called the elementary body (EB), exhibits minimal metabolic activity and promotes its entry into human epithelial cells. Once inside the host cell, Chlamydia EBs reside within a membrane-bound compartment called the inclusion. EBs then rapidly differentiate into a replicative form, called the reticulate body (RB), which is metabolically active and noninfectious. As RBs replicate by binary fission, they synthesize effector proteins that remodel and hijack the host cell to promote their survival (6). In doing so, Chlamydia subverts many aspects of the host cellular environment, including the host cytoskeleton, a critical element of successful infection and dissemination of C. trachomatis within the host.

EBs initially invade the host cell by attaching to the plasma membrane and injecting the chlamydial protein Tarp (translocated actin recruiting protein) into the host cytoplasm via the chlamydial type III secretion system. Phosphorylation of Tarp in the cytosol allows Tarp to recruit the guanine nucleotide exchange factors (GEFs) Sos1 and Vav2, which activate Rac1 (7). Tarp directly binds actin monomers to nucleate new linear filaments (8, 9). Subsequently, the Arp2/3 complex is activated, and branched actin filaments are polymerized underneath the bacterial binding site to promote the entry of the EBs into the nascent inclusion (10–12).

The second wave of cytoskeletal manipulation begins around 16 h postinfection (hpi), when MTs are assembled around the inclusion (13). These scaffolds are subsequently stabilized and undergo posttranslational modification (PTM), including detyrosination and acetylation (14, 15). PTMs influence MT structure and depolymerization rates, further increasing their stability (16, 17). We have previously established that the small GTPase ARF1 controls the posttranslational modification of MT scaffolds. PTM-MT scaffolds then position Golgi ministacks around the inclusion, which provides lipids to facilitate the rapid expansion of the inclusion (14, 15, 18). Around 32 hpi, actin scaffolds form around the inclusion. These structures physically reinforce the inclusion membrane to prevent premature lysis, as the inclusion expands to occupy most of the host cytoplasm (19). Using actin-depolymerizing agents and small interfering RNA (siRNA), the formation of F-actin at the inclusion was shown to be RhoA dependent (19).

Finally, Chlamydia exits its host cell either by controlled cell lysis or through extrusion, which also relies on the intervention of the host cytoskeleton. Extrusion is a nonlytic process during which the inclusion protrudes out of the infected cell before pinching off the plasma membrane (20). This event requires the mobilization of RhoA, WASP, and myosin II. Interestingly, MT depolymerization with nocodazole does not affect extrusion, suggesting this event is actin specific (20).

Interference with the host cytoskeleton dramatically impacts the outcome of Chlamydia infection. The inhibition of PTM-MT scaffolds decreases Chlamydia infectivity, whereas their enhancement increases it, demonstrating the importance of this cytoskeletal element in Chlamydia development (14). Global inhibition of actin polymerization with latrunculin also causes early rupture of inclusions, indicating that actin is essential for maintaining inclusion stability (19). Inclusion stability solely depends on actin, as MT depolymerization does not impact inclusion integrity (19).

Previous work investigating Chlamydia has been significantly limited by its reliance on cytoskeleton-modulating drugs. These drugs disrupt the host cytoskeleton in its entirety and often indirectly impact other cytoskeletal elements (21). As a result, elucidating the precise local role of actin and MT scaffolds during Chlamydia infection has been challenging. However, with advances in Chlamydia genetics, targeted disruption of Chlamydia’s virulence system and its effector proteins is now possible without indirectly affecting native host processes. Recently, we and others showed that a single chlamydial effector, InaC (also called CT813 or CTL0184), controls the formation of the kinetically and functionally distinct actin and PTM-MT scaffolds (15, 19, 22). InaC knockout (KO) Chlamydia produces less-infectious progeny and generates smaller inclusions, indicating that InaC-dependent pathways are essential for Chlamydia fitness (15, 19). We also demonstrated that InaC directly interacts with the host small GTPases ARF1 and ARF4 to control the formation of PTM-MTs and, consequently, the dispersal of Golgi ministacks around the inclusion during infection (15). This event occurs midcycle at 16 to 24 hpi. In addition to InaC, it has been shown that actin scaffold formation around 32 hpi is mainly dependent on the host GTPase RhoA (19). Whether InaC regulates RhoA and how both GTPases are coordinated during infection to generate their specific scaffolds at the optimal time is unknown.

Here, we report that while RhoA can be recruited to the inclusion in the absence of InaC, its activation, which leads to the formation of actin scaffolds, requires InaC. Furthermore, we discovered that the presence of RhoA downregulates ARF1 activation, while ARF1-GTP inhibits RhoA activation, highlighting cross talk between two small GTPases to coordinate PTM-MT and actin scaffolds during infection. Overall, our findings establish that a single chlamydial effector, InaC, is a master regulator of actin and MT dynamics during Chlamydia infection.

## RESULTS

### RhoA activation is required for the formation of actin scaffolds around the inclusion.

To understand the exact role of RhoA during C. trachomatis infection and to assess whether RhoA is the sole regulator of actin scaffold formation, we generated CRISPR/Cas9 RhoA KO HeLa cells (see Fig. S1A in the supplemental material). HeLa cells transfected with the Cas9 vector without guide RNA were used as a control. RhoA KO cells also express similar amounts of actin, α-tubulin, and acetylated α-tubulin compared to control cells (Fig. S1B). We detected a modest but statistically insignificant increase in detyrosinated α-tubulin in the absence of RhoA. As expected, RhoA KO cells are viable and divide similarly to control cells (Fig. S1C and D).

10.1128/mBio.02397-21.1 FIG S1 Generation of the RhoA KO CRISPR/Cas9 cell line. (A) Control and RhoA KO cells were lysed and analyzed by Western blotting using the indicated antibodies. HSP70 was used as a control. (B) Quantification of relative band intensities of the Western blots in panel A. The graph represents the average ratio of each cytoskeletal element on the *x* axis to the HSP70 loading control from 3 independent experiments ± the standard deviation. Data are normalized to the empty vector control cell line. ns, not significant. (C and D) CRISPR/Cas9 RhoA KO (C) and control HeLa (D) cells were stained with CFSE and incubated at 37°C for 24, 48, 72, and 96 h. At each time point, the intensity of CFSE remaining for each sample was measured by flow cytometry. Download FIG S1, PDF file, 0.7 MB. Copyright © 2021 Haines et al. 2021 Haines et al. https://creativecommons.org/licenses/by/4.0/ This content is distributed under the terms of the Creative Commons Attribution 4.0 International license.

**FIG 1:**
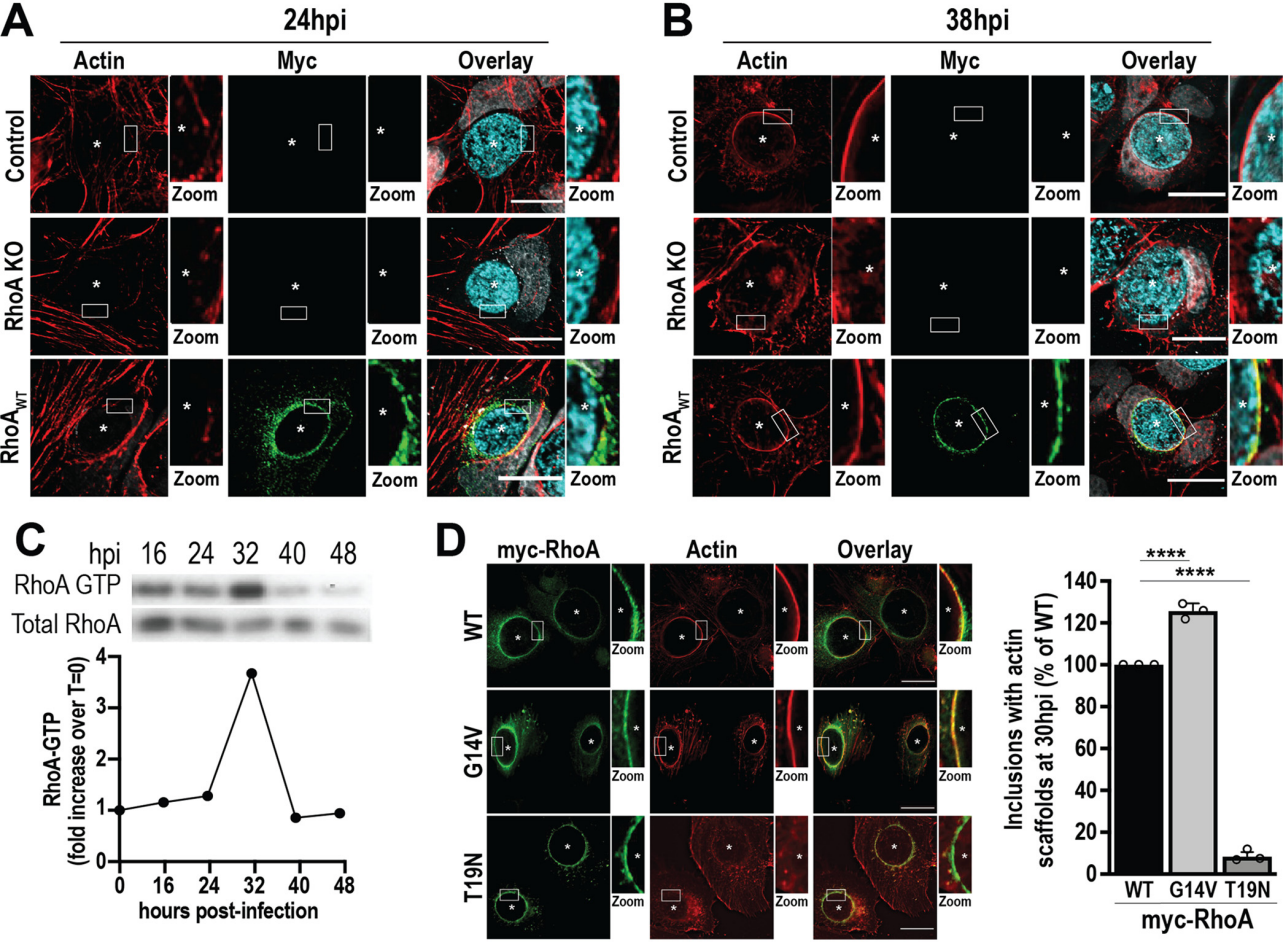
Active RhoA is required for the formation of actin scaffolds around the inclusion. (A and B) Control and RhoA KO cells were infected with WT C. trachomatis L2 (MOI of 2) and transfected with empty or myc-RhoA_WT_ DNA at 4 hpi. Cells were fixed at 24 or 38 hpi and labeled with phalloidin to label actin (red), anti-myc antibody to label RhoA (green), and anti-MOMP antibody to label individual Chlamydia (cyan). Asterisks denote inclusions. The white box indicates the magnified area shown to the right of the image (Zoom). Images are representative of 3 independent experiments. Scale bar, 25 μm. (C) HeLa cells were infected with WT C. trachomatis L2 (MOI of 1) for the indicated times prior to lysis and isolation of RhoA-GTP using the RhoA binding domain of Rhotekin immobilized on agarose beads. In the Western blot, RhoA-GTP denotes the RhoA signal from the pulldown, and Total RhoA represents the amount of RhoA from the cell lysate. The graph indicates the fold increase in RhoA-GTP compared to a noninfected control (*t* = 0). Results are representative of two independent experiments. (D) RhoA KO cells were infected with WT C. trachomatis L2 (MOI of 2) and transfected with myc-RhoA_WT_, myc-RhoA_G14V_, or myc-RhoA_T19N_ DNA at 4 hpi. Cells were fixed 30 hpi and labeled with anti-myc (green) antibody and phalloidin (red). Scale bar, 20 μm. Asterisks denote inclusions. The white box indicates the magnified area shown to the right of the image (Zoom). The graph denotes the average percentage of inclusions containing actin scaffolds from 3 independent experiments ± the standard deviation. Data are normalized to myc-RhoA_WT_-expressing cells. A minimum of 100 inclusions was counted for each condition per experiment. ****, *P* < 0.0001.

Next, we assessed the kinetics of actin scaffold formation in the presence and absence of RhoA during Chlamydia infection. Control and RhoA KO cells were infected with wild-type (WT) C. trachomatis L2 prior to the analysis of actin scaffolds around the inclusion at different times postinfection. In parallel, RhoA KO cells were transfected with a vector encoding myc-RhoA_WT_ (RhoA_WT_) at 3 to 4 hpi to restore RhoA expression. In control cells, actin scaffolds are detected at 38 hpi, but not 24 hpi (Fig. 1A and B, Control), consistent with previous observations that actin scaffolds form late during infection (19). In contrast, actin scaffolds were not detectable at either time point in RhoA KO cells (Fig. 1A and B, RhoA KO). The expression of myc-RhoA_WT_ rescued the loss of actin scaffolds in RhoA KO cells at 38 hpi (Fig. 1B, RhoA_WT_), indicating that the loss of actin scaffolds in RhoA KO cells is not due to an off-target effect of Cas9.

Like all small GTPases, RhoA cycles between GDP- and GTP-bound states, which in turn control its interactions with downstream effectors (23). To assess whether Chlamydia infection influences the activation state of RhoA to promote actin polymerization around the inclusion, we infected HeLa cells with C. trachomatis L2 and generated lysates from mid to late times postinfection (16 to 48 hpi). The levels of RhoA-GTP were then analyzed using the RhoA-binding domain (RBD) of the effector Rhotekin (24). The activation of RhoA reaches a maximal level at 32 hpi (Fig. 1C), which correlates with both the formation of actin scaffolds and the recruitment of endogenous RhoA to the inclusion (Fig. S2). Note that RhoA activation is a transient event, as it is deactivated and returns to noninfected levels at ∼40 hpi. These data indicate that C. trachomatis triggers the activation of RhoA at the same time that actin polymerization occurs around the inclusion.

To establish the role of RhoA activation in the formation of actin scaffolds, we exploited the well-characterized G14V (myc-RhoA_G14V_, GTP locked) and T19N (myc-RhoA_T19N_, dominant negative) RhoA mutants, which have been used extensively to explore the biological activities of RhoA (25–27). At 3 to 4 hpi with C. trachomatis L2, we transfected RhoA KO cells with either myc-RhoA_G14V_ or myc-RhoA_T19N_ and compared the formation of actin scaffolds to that of cells transfected with myc-RhoA_WT_. We found that myc-RhoA_G14V_, but not myc-RhoA_T19N_, supports actin scaffold formation (Fig. 1D). Compared to infected myc-RhoA_WT_-expressing cells, the expression of myc-RhoA_G14V_ maximizes actin scaffold formation to 126% ± 3.7%. In comparison, only 9% ± 2.1% of inclusions displayed actin scaffolds in RhoA_T19N_-expressing cells, demonstrating that active RhoA is required to promote actin scaffold formation. Interestingly, actin scaffolds become detectable at 24 hpi in cells expressing constitutively active myc-RhoA_G14V_, while they are absent in cells expressing myc-RhoA_WT_ and myc-RhoA_T19N_ (see Fig. S3 in the supplemental material). In this case, we observe that 86% ± 4.2% of inclusions have actin scaffolds as early as 24 hpi. This is not due to a shift in RhoA recruitment since endogenous RhoA is already present on the inclusion at 24 hpi (Fig. S2) despite the absence of actin scaffolds (Fig. 1A). Altogether, these data indicate that the temporal activation of RhoA is critical and dictates the kinetics of actin scaffold formation during infection.

10.1128/mBio.02397-21.2 FIG S2 Endogenous RhoA is recruited to the inclusion with specific kinetics. (A) HeLa cells were infected with WT C. trachomatis L2 (MOI of 2) for 32 h. The cells were fixed and labeled with anti-RhoA (red) and anti-IncA (green) antibodies. IncA staining was used to delimit the inclusion membrane. Scale bar, 10 μm. The line intensity scans (right) indicate the coincidence of RhoA with IncA staining. RhoA is denoted as red lines, and IncA is shown as green lines. (B) HeLa cells were infected with WT C. trachomatis L2 (MOI of 2), fixed at 16 to 48 hpi, and labeled with anti-RhoA (red) and anti-IncA (green) antibodies. DNA was labeled with Hoechst (gray). Scale bar, 30 μm. The boxes indicate a representative inclusion for each time point that is magnified on the left. Scale bar, 10 μm. (C) The graph depicts the average percentage of inclusions with RhoA on the inclusion membrane from 3 independent experiments ± the standard deviation. A minimum of 100 inclusions was counted for each condition per experiment. Download FIG S2, PDF file, 4.8 MB. Copyright © 2021 Haines et al. 2021 Haines et al. https://creativecommons.org/licenses/by/4.0/ This content is distributed under the terms of the Creative Commons Attribution 4.0 International license.

10.1128/mBio.02397-21.3 FIG S3 Expression of constitutively active RhoA is sufficient to promote the early formation of actin scaffolds. (A) RhoA KO HeLa cells were infected with WT C. trachomatis L2 (MOI of 2) and transfected with myc-RhoA_WT_, myc-RhoA_G14V_, or myc-RhoA_T19N_ DNA at 4 hpi. Cells were then fixed at 24 hpi and labeled with anti-myc antibody (green) and phalloidin (red). Asterisks denote inclusions. The white box indicates the magnified area to the right of the image (zoom). Scale bar, 20 μm. (B) The graph depicts the average percentage of inclusions with actin scaffolds in cells transfected with the indicated myc-RhoA DNA from 3 independent experiments ± the standard deviation. A minimum of 100 inclusions was counted for each condition per experiment. ****, *P* < 0.0001. Download FIG S3, PDF file, 3.0 MB. Copyright © 2021 Haines et al. 2021 Haines et al. https://creativecommons.org/licenses/by/4.0/ This content is distributed under the terms of the Creative Commons Attribution 4.0 International license.

### The chlamydial effector InaC is required for the activation of RhoA.

**FIG 2:**
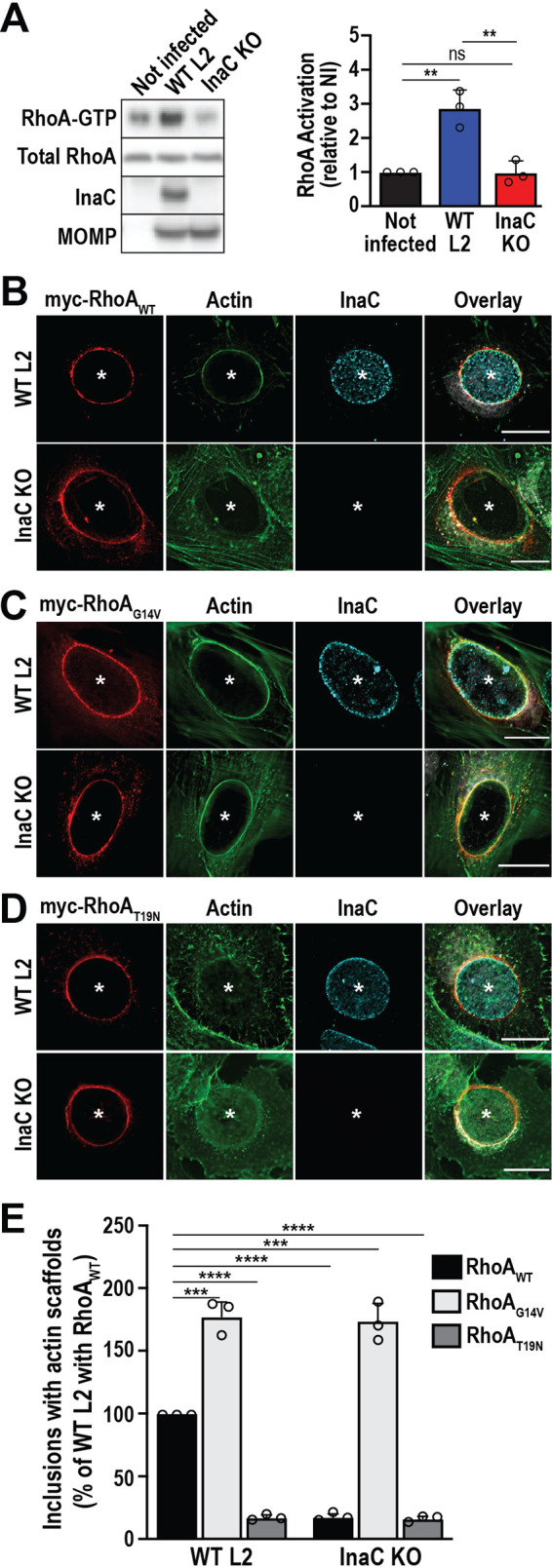
The chlamydial effector InaC is required for the activation of RhoA. (A) HeLa cells were infected with the indicated C. trachomatis L2 strains (MOI of 1) for 32 h. RhoA-GTP was isolated as described in Fig. 1C. In the Western blot, anti-InaC antibody confirms the loss of InaC in the InaC KO strain and anti-MOMP antibody confirms infection. The graph denotes the average fold change in RhoA-GTP compared to the uninfected control ± the standard deviation from 3 independent experiments. **, *P* < 0.01; ns, not significant. (B to D) RhoA KO cells were infected with WT or InaC KO C. trachomatis L2 (MOI of 2) and transfected with myc-RhoA_WT_ (B), myc-RhoA_G14V_ (C), or myc-RhoA_T19N_ (D) DNA at 4 hpi. Cells were fixed at 38 hpi and labeled with phalloidin (green), anti-myc antibody (red), and anti-InaC antibody (cyan). Asterisks denote inclusions. Scale bar, 20 μm. Results are representative of at least 3 independent experiments. (E) The graph displays the average percentage of inclusions with actin scaffolds ± the standard deviation from 3 independent experiments. Data are normalized to cells infected with WT C. trachomatis L2 and transfected with myc-RhoA_WT_ DNA. A minimum of 100 inclusions was counted for each condition per experiment. ***, *P* < 0.001; ****, *P* < 0.0001.

InaC is a chlamydial effector that is required for the formation of actin scaffolds during Chlamydia infection (15, 19). To establish whether InaC regulates the activity of RhoA during infection, we made use of a C. trachomatis L2 mutant in which the InaC gene was inactivated using Targetron (InaC KO Chlamydia) (15). We then infected HeLa cells with InaC KO C. trachomatis L2 and quantified the amount of RhoA-GTP at 32 hpi. Infection with WT C. trachomatis L2 increased RhoA-GTP levels 2.9 ± 0.54-fold compared to uninfected control cells (Fig. 2A). In contrast, InaC KO-infected cells exhibited RhoA-GTP levels similar to control cells (0.983 ± 0.34-fold change), indicating that InaC is required in RhoA activation during C. trachomatis infection.

One way that proteins regulate each other is through direct interactions. We thus tested whether InaC forms a complex with RhoA to control its activation locally. To do so, we conducted a series of immunoprecipitation experiments in the presence and absence of infection in different configurations. However, we could not detect an interaction (see Fig. S4 in the supplemental material), indicating that either InaC and RhoA do not bind or the interaction is too weak or transient to detect.

10.1128/mBio.02397-21.4 FIG S4 RhoA does not interact with InaC. (A) HeLa cells were infected with WT C. trachomatis L2 (MOI of 5) and transfected with myc-RhoA_WT_ DNA at the time of infection. myc-RhoA was immunoprecipitated from cell lysates at 32 hpi using the mouse anti-myc antibody. Normal mouse IgG was used as an immunoprecipitation control (Ctrl IgG). Immunoprecipitates and cell lysates were analyzed by Western blotting using anti-myc, anti-InaC, and anti-HSP70 antibodies. HSP70 was used as a loading control. (B) HeLa cells were infected with InaC-FLAG-overexpressing C. trachomatis L2 (MOI of 5) and treated with 5 ng/ml aTc 4 hpi to induce InaC-FLAG expression. InaC-FLAG was immunoprecipitated from cell lysates at 32 hpi using a mouse anti-FLAG antibody. Normal mouse IgG was used as an immunoprecipitation control (Ctrl IgG). Immunoprecipitates and cell lysates were analyzed by Western blotting using anti-FLAG, anti-ARF1, anti-RhoA, and anti-HSP70 antibodies. Anti-ARF1 was used as a positive binding control because it interacts with InaC (15). HSP70 was used as a loading control. (C) HeLa cells were infected with InaC-FLAG-overexpressing C. trachomatis L2 (MOI of 5) and cotransfected with ARF1_WT_-HA and either empty myc vector (myc), myc-RhoA_WT_, myc-RhoA_G14V_, or myc-RhoA_T19N_ DNA at the time of infection. InaC-FLAG expression was induced with 5 ng/ml aTc 4 hpi. InaC-FLAG was immunoprecipitated from cell lysates at 24 and 40 hpi using mouse anti-FLAG antibody. Normal mouse IgG was used as an immunoprecipitation control (Ctrl IgG). Immunoprecipitates and cell lysates were analyzed by Western blotting using anti-FLAG, anti-HA, anti-myc, and anti-HSP70 antibodies. HSP70 was used as a loading control. (D) Samples were prepared and analyzed as in panel C, but cells were cotransfected with myc-RhoA_WT_ and either empty HA vector (HA), ARF1_WT_-HA, ARF1_Q71L_-HA, or ARF1_T31N_-HA DNA at the time of infection. (E) HeLa cells were infected with InaC KO C. trachomatis L2 for 32 h and cotransfected with FLAG-InaC DNA and either ARF1_Q71L_-HA or ARF1_T31N_-HA DNA at the time of infection. FLAG-InaC was immunoprecipitated from cell lysates using an anti-FLAG antibody. Immunoprecipitates and cell lysates were analyzed by Western blotting using anti-FLAG, anti-RhoA, anti-HA, and anti-HSP70 antibodies. HSP70 was used as a loading control. (F) HeLa cells were cotransfected with FLAG-InaC, myc-RhoA_WT_, and either empty HA vector (HA), ARF1_WT_-HA, ARF1_Q71L_-HA, or ARF1_T31N_-HA DNA for 24 h. InaC-FLAG was immunoprecipitated using an anti-FLAG antibody. Immunoprecipitates and cell lysates were analyzed by Western blotting using anti-FLAG, anti-HA, anti-myc, and anti-HSP70 antibodies. HSP70 was used as a loading control. Download FIG S4, PDF file, 5.7 MB. Copyright © 2021 Haines et al. 2021 Haines et al. https://creativecommons.org/licenses/by/4.0/ This content is distributed under the terms of the Creative Commons Attribution 4.0 International license.

Even though InaC and RhoA do not form a detectable complex, the recruitment of RhoA to the inclusion could still be indirectly impacted by the absence of InaC, which would explain the reduction in RhoA-GTP in cells infected with InaC KO C. trachomatis L2 (Fig. 2A). To assess whether RhoA recruitment to the inclusion depends on InaC, we used RhoA KO cells complemented with myc-RhoA_WT_ and infected with WT or InaC KO C. trachomatis L2. At 32 to 38 hpi, infected cells were fixed and stained with anti-myc and anti-InaC antibodies to label RhoA and the inclusion membrane, respectively. As expected, myc-RhoA_WT_ was present on the inclusion membrane in WT C. trachomatis L2-infected cells (Fig. 2B, WT L2). Interestingly, myc-RhoA_WT_ was also detected on the inclusion membrane of InaC KO C. trachomatis L2-infected cells (Fig. 2B, InaC KO), indicating that RhoA recruitment is InaC independent. Next, we assessed whether both mutant forms of RhoA are differentially recruited during infection with InaC KO Chlamydia. As shown in Fig. 2C and D, myc-RhoA_G14V_ (Fig. 2C) and myc-RhoA_T19N_ (Fig. 2D) are also recruited to the inclusion in both WT and InaC KO C. trachomatis L2-infected cells. These data further indicate that RhoA is recruited to the inclusion independently of its activation state.

We then determined whether the loss of actin scaffolds in InaC KO C. trachomatis L2-infected cells is due to a defect in RhoA activation. To do so, we infected RhoA KO HeLa cells with WT or InaC KO C. trachomatis L2, followed by transfection with myc-RhoA_WT_, myc-RhoA_G14V_, or myc-RhoA_T19N_ DNA. The cells were fixed at 32 to 38 hpi and labeled with anti-myc and anti-InaC antibodies. As observed earlier, myc-RhoA_WT_ and myc-RhoA_G14V_, but not myc-RhoA_T19N_, promote the formation of actin scaffolds during WT C. trachomatis infection (Fig. 2B to E, WT L2). Strikingly, the expression of myc-RhoA_G14V_, but not myc-RhoA_WT_ or myc-RhoA_T19N_, rescues the InaC KO phenotype and fully restores the formation of actin scaffolds (Fig. 2B to E, InaC KO), indicating that the loss of actin scaffolds in InaC KO-infected cells is due to an inability to activate RhoA. Altogether, these results demonstrate that InaC is not required to recruit RhoA but rather to locally activate it and promote actin scaffold formation around the inclusion. Since RhoA does not interact with InaC, it is likely that an additional host or bacterial factor that remains to be identified is involved.

### InaC-RhoA-mediated cytoskeletal scaffolds contribute to inclusion stability.

**FIG 3:**
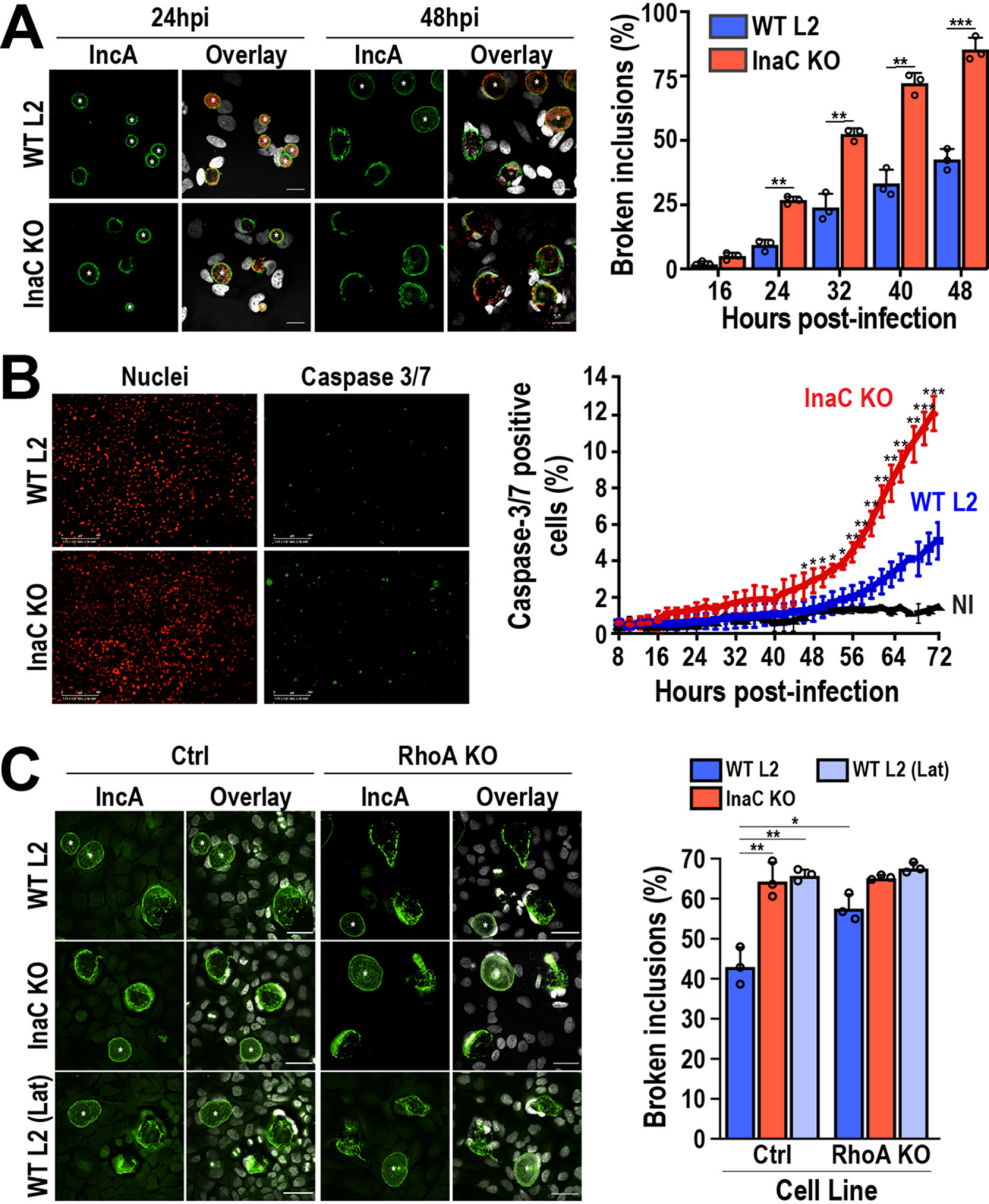
InaC-mediated cytoskeletal pathways contribute to the stability of chlamydial inclusions. (A) HeLa cells were infected with WT or InaC KO C. trachomatis L2 (MOI of 0.5) and fixed at the indicated times postinfection. Cells were labeled with anti-IncA antibody (green) to label the inclusion membrane and anti-MOMP antibody (red). Hoechst was used to label DNA (gray). Asterisks denote intact inclusions. Scale bar, 10 μm. The adjacent graph represents the average percentage of broken inclusions at the indicated time points from 3 independent experiments ± the standard deviation. **, *P* < 0.01; ***, *P* < 0.001. (B) Nuclight Red HeLa cells were infected with WT or InaC KO C. trachomatis L2 (MOI of 1) for 6 h before the addition of IncuCyte caspase 3/7 green dye for apoptosis at a final concentration of 5 μM. The cells were then imaged every 2 h until 72 hpi on an IncuCyte Zoom imaging system. Nuclei of HeLa cells are shown in red, and caspase 3/7 puncta are shown in green. Scale bar, 300 μm. The graph depicts the average percentage of caspase 3/7-positive cells from 4 independent experiments ± the standard deviation. A minimum of 100 inclusions was counted for each condition. NI, not-infected cells. *, *P* < 0.05; **, *P* < 0.01; ***, *P* < 0.001. (C) Control and RhoA KO HeLa cells were infected with WT or InaC KO C. trachomatis L2 (MOI of 0.5) and fixed at 72 hpi. As a control, one set of WT C. trachomatis L2-infected cells was treated with 0.5 μM latrunculin B or ethanol (vehicle control) in cell culture medium for 30 min at 37°C 5 h prior to fixation. Vehicle and latrunculin B were washed out with cell culture medium, and the cells were fixed at 72 hpi. Cells were labeled with anti-IncA antibody (green) to label the inclusion membrane and Hoechst (gray) to stain DNA. Scale bar, 40 μm. Asterisks denote intact inclusions. WT L2 (Lat), latrunculin B-treated cells. The graph depicts the average percentage of broken inclusions from 3 independent experiments ± the standard deviation. A minimum of 100 inclusions was counted for each condition. *, *P* < 0.05; **, *P* < 0.01; ***, *P* < 0.001.

InaC is required for the formation of both PTM-MT and actin scaffolds (15, 22). However, their direct contribution to inclusion stability is not well understood. To test the importance of these scaffolds in Chlamydia fitness, we infected HeLa cells with InaC KO C. trachomatis L2 and assessed inclusion stability over time. In WT C. trachomatis L2-infected cells, we observed that ∼40% of inclusions were ruptured at 48 hpi. In contrast, ∼50% of the InaC KO inclusions were lysed by 32 hpi (Fig. 3A), indicating a substantial shift in inclusion stability in the absence of InaC. This early release of Chlamydia into the cytosol correlated with a 2.4-fold increase in cell death measured by caspase 3/7 activation. While only 5% ± 0.98% of WT C. trachomatis L2-infected cells were positive for caspase 3/7 activation, 12% ± 0.86% of InaC KO C. trachomatis L2-infected cells were positive at 72 hpi (Fig. 3B). InaC KO inclusions were stabilized when InaC-FLAG was reintroduced on a plasmid (see Fig. S5A and C in the supplemental material). As expected, the complementation of InaC expression also rescued the loss of actin scaffolds (Fig. S5B). Overall, this indicates that InaC is critical for the maintenance of inclusion stability.

10.1128/mBio.02397-21.5 FIG S5 Complementation of InaC KO with InaC-FLAG rescues the actin scaffold and inclusion lysis phenotype of InaC KO C. trachomatis. (A) HeLa cells were infected with InaC-FLAG-expressing InaC KO C. trachomatis L2 (MOI of 2), and InaC-FLAG expression was induced with 5 ng/ml aTc 4 hpi. Cells were fixed at 32 hpi and labeled with anti-FLAG (green) and anti-MOMP (red) antibodies. DNA was labeled with Hoechst (gray). Asterisks denote inclusions. Scale bar, 20 μm. (B) HeLa cells were infected with InaC-FLAG-expressing InaC KO C. trachomatis L2 (MOI of 2), and InaC-FLAG expression was induced with 5 ng/ml aTc 4 hpi. A set of infected cells were treated with dimethyl sulfoxide (DMSO) (0 ng/ml aTc) as a negative control. Cells were fixed at 32 hpi and labeled with phalloidin (red) and anti-MOMP (red) antibodies. DNA was labeled with Hoechst (gray). Asterisks denote inclusions. Scale bar, 20 μm. The boxes represent enlarged (Zoom) images of inclusions to show the presence of actin scaffolds in InaC-FLAG-expressing (5 ng/ml aTc) InaC KO C. trachomatis L2. (C) HeLa cells were infected with WT or InaC-FLAG-expressing InaC KO C. trachomatis L2 (MOI of 0.5), and InaC-FLAG expression was induced with 5 ng/ml aTc 4 hpi. Cells were fixed 48 hpi and labeled with anti-IncA (green) and anti-MOMP (red) antibodies. DNA was labeled with Hoechst (gray). Asterisks denote intact inclusions. Scale bar, 20 μm. The graph represents the average percentage of intact inclusions normalized with WT C. trachomatis L2 from 3 independent experiments ± the standard deviation. A minimum of 100 inclusions was counted for each condition per experiment. Download FIG S5, TIF file, 2.6 MB. Copyright © 2021 Haines et al. 2021 Haines et al. https://creativecommons.org/licenses/by/4.0/ This content is distributed under the terms of the Creative Commons Attribution 4.0 International license.

To specifically test the impact of the loss of actin scaffolds on inclusion stability, RhoA KO cells were infected with WT C. trachomatis L2, and inclusion lysis was measured at 72 hpi as it corresponds to maximal caspase 3/7 activation (Fig. 3B). As controls, we infected RhoA KO cells with InaC KO C. trachomatis L2 and treated WT C. trachomatis L2-infected cells with latrunculin B to depolymerize all host cell actin. Consistent with previous reports (19), latrunculin B treatment increased levels of inclusion lysis [Fig. 3C, WT L2, (Lat)]. Compared to control cells, inclusion lysis increased by ∼15% in the absence of RhoA (Fig. 3C, Ctrl-WT L2 versus RhoA KO-WT L2). Control and RhoA KO cells infected with InaC KO C. trachomatis L2 showed similar levels of inclusion lysis to RhoA KO cells infected with WT C. trachomatis L2, indicating that InaC and RhoA function in the same pathway to control inclusion stability (Fig. 3C, Ctrl InaC KO versus RhoA KO WT L2). These results suggest that actin scaffolds, but not PTM-MT scaffolds, generated through an InaC-RhoA-dependent pathway specifically contribute to the stability of inclusions during infection.

### The presence of RhoA inhibits the formation of PTM-MT scaffolds during infection.

PTM-MT scaffolds around the inclusion are most evident around 24 hpi, whereas the formation of actin scaffolds around the inclusion begins later, around 32 hpi (15, 19). Furthermore, actin scaffolds do not form in the absence of RhoA, and PTM-MT scaffolds are disorganized in the absence of ARF1. These data suggest that these two cytoskeletal pathways are independent of each other and are controlled by their corresponding small GTPase. This concept is supported by the fact that each GTPase is activated at specific times during infection: 16 hpi for ARF1 (see Fig. S6 in the supplemental material) and 32 hpi for RhoA (Fig. 1C). Yet, the requirement for the chlamydial protein InaC to control both cytoskeletal events indicates that some degree of cross talk likely exists between these two pathways. Since the inclusion is a fundamental component of Chlamydia pathogenicity, this cross talk may allow InaC to finely control the activation/inactivation of each pathway to appropriately time Golgi repositioning and inclusion stability. In this case, we would expect that altering the expression level and/or activity level of either GTPase would impact the formation of the other cytoskeletal element.

10.1128/mBio.02397-21.6 FIG S6 ARF1 is maximally activated at 16 hpi. HeLa cells were infected with WT C. trachomatis L2 (MOI of 5), and lysates were prepared 8 to 48 hpi. Noninfected lysates were prepared as controls. ARF1-GTP in the lysates was isolated using GST-GGA1 bound to glutathione agarose. Total ARF1 from the starting material (cell lysate) was used as a loading control. Download FIG S6, PDF file, 0.10 MB. Copyright © 2021 Haines et al. 2021 Haines et al. https://creativecommons.org/licenses/by/4.0/ This content is distributed under the terms of the Creative Commons Attribution 4.0 International license.

**FIG 4:**
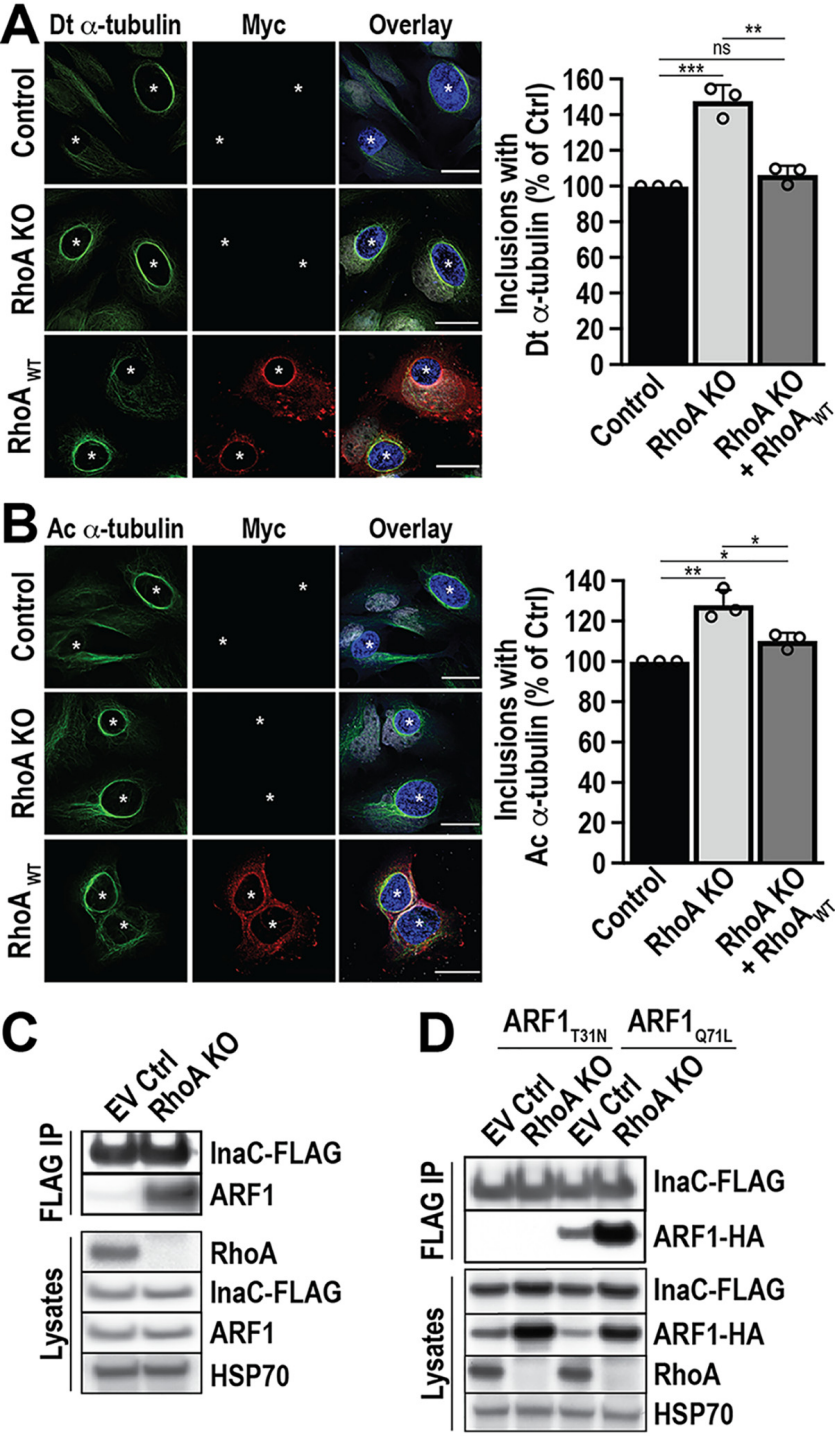
The formation of PTM-MT scaffolds during infection is enhanced in the absence of RhoA. (A and B) Control and RhoA KO HeLa cells were infected with WT C. trachomatis L2 (MOI of 2) and transfected with empty vector or myc-RhoA_WT_ DNA at 4 hpi. Cells were fixed at 24 hpi and labeled with anti-detyrosinated α-tubulin (Dt α-tubulin) (A, green), anti-acetylated α-tubulin (Ac α-tubulin) (B, green), anti-myc (red), and anti-MOMP (blue) antibodies. Scale bar, 30 μm. The graphs represent the average percentage of inclusions with Dt α-tubulin (A) or Ac α-tubulin (B) scaffolds normalized to infected control HeLa cells. A minimum of 100 inclusions was counted for each condition per experiment. *, *P* < 0.05; **, *P* < 0.01; ***, *P* < 0.001; ns, not significant. (C) Control and RhoA KO HeLa cells were infected with InaC-FLAG-overexpressing C. trachomatis L2 (MOI of 5) and treated with 5 ng/ml anhydrotetracycline (aTc) 4 hpi to induce InaC-FLAG expression. Cells were lysed at 24 hpi, and InaC-FLAG was immunoprecipitated using an anti-FLAG antibody. ARF1 binding was assessed with anti-ARF1 antibody. Lysates were analyzed as a control. Anti-RhoA antibody confirms the loss of RhoA in the RhoA KO. HSP70 served as a loading control. Results are representative of 2 independent experiments. (D) Control and RhoA KO HeLa cells were infected with InaC-FLAG-overexpressing C. trachomatis L2 (MOI of 5) and transfected with ARF1_T31N_-HA or ARF1_Q71L_-HA at 4 hpi. InaC-FLAG expression was induced with 5 ng/ml aTc 4 hpi. Cells were lysed at 24 hpi, and InaC-FLAG was immunoprecipitated using anti-FLAG antibody. ARF1 binding was assessed with anti-HA antibody. Lysates were analyzed as a control. Anti-RhoA antibody confirms the loss of RhoA in the RhoA KO. HSP70 served as a loading control. Results are representative of 2 independent experiments.

To test this hypothesis, we first investigated the impact of RhoA depletion on the formation of PTM-MTs during infection. RhoA KO cells were infected with WT C. trachomatis L2, and the PTM-MT scaffolds around the inclusion at 24 hpi were monitored using immunofluorescence microscopy. As expected, PTM-MT scaffolds still assemble around the inclusion in the absence of RhoA, demonstrating that RhoA is not required for their formation (Fig. 4A and B). However, we observe increases of 48% ± 8.8% and 28% ± 7.4% of detyrosinated and acetylated α-tubulin scaffolds, respectively, compared to control cells (Fig. 4A and B, graphs). This enhanced formation of PTM-MT scaffolds is suppressed to WT levels when myc-RhoA_WT_ is expressed in RhoA KO cells, confirming that the phenotype is specifically due to the loss of RhoA (Fig. 4A and B, RhoA_WT_). Furthermore, the expression of either active or inactive RhoA mutants in RhoA KO cells suppresses the enhanced formation of PTM-MTs similarly to myc-RhoA_WT_ (see Fig. S7 in the supplemental material). These results indicate that this phenomenon is due to the presence of RhoA rather than a specific nucleotide-bound state. Altogether, these data suggest that the absence of RhoA enhances the formation of PTM-MTs.

10.1128/mBio.02397-21.7 FIG S7 The formation of PTM-MTs is insensitive to the nucleotide-bound state of RhoA. (A and B) RhoA KO HeLa cells were infected with WT C. trachomatis L2 (MOI of 2) and transfected with myc-RhoA_WT_, myc-RhoA_G14V_, or myc-RhoA_T19N_ DNA at 4 hpi. Cells were fixed 24 hpi and labeled with anti-myc (green), anti-detyrosinated α-tubulin (Dt α-tubulin) (A, red), or anti-acetylated α-tubulin (Ac α-tubulin) (B, red) antibodies. Scale bar, 20 μm. Asterisks denote inclusions. The white box denotes the magnified area to the right of the image (Zoom). The graphs represent the average percentage of inclusions with detyrosinated α-tubulin (A) or acetylated α-tubulin (B) scaffolds compared to cells expressing myc-RhoA_WT_ from 3 independent experiments ± the standard deviation. A minimum of 100 inclusions was counted for each condition per experiment. Download FIG S7, PDF file, 15.4 MB. Copyright © 2021 Haines et al. 2021 Haines et al. https://creativecommons.org/licenses/by/4.0/ This content is distributed under the terms of the Creative Commons Attribution 4.0 International license.

Since ARF1 is a critical factor in forming PTM-MT scaffolds (15), the enhancement of PTM-MT scaffolds in the RhoA KO could be due to increased complex formation between InaC and ARF1 and/or increased ARF1 activation. To test both possibilities, we infected RhoA KO cells with C. trachomatis L2 expressing InaC-FLAG. We then assessed the interactions between InaC-FLAG and endogenous ARF1 compared to the complex formed in control cells. As shown in Fig. 4C, we observe a striking increase in ARF1 binding to InaC-FLAG in the absence of RhoA at 24 hpi. Next, we tested whether the enhanced interaction between ARF1 and InaC is with active or inactive ARF1. Control and RhoA KO cells were transfected with either GDP-bound (ARF1_T31N_-HA [hemagglutinin]) or GTP-bound (ARF1_Q71L_-HA) ARF1 (15, 28) at 3 to 4 hpi with C. trachomatis L2 expressing InaC-FLAG. InaC was immunoprecipitated using an anti-FLAG antibody, and the immunoprecipitates were analyzed by Western blotting to identify which ARF1 mutant was bound. As shown in Fig. 4D, only ARF1_Q71L_-HA binds to InaC. The interaction with ARF1-Q71L and InaC is enhanced in the absence of RhoA, demonstrating that the lack of RhoA results in an increase in InaC:ARF1-GTP binding and consequently PTM-MT scaffold formation.

### Activation of ARF1 impairs RhoA-GTP-dependent actin scaffold formation.

**FIG 5:**
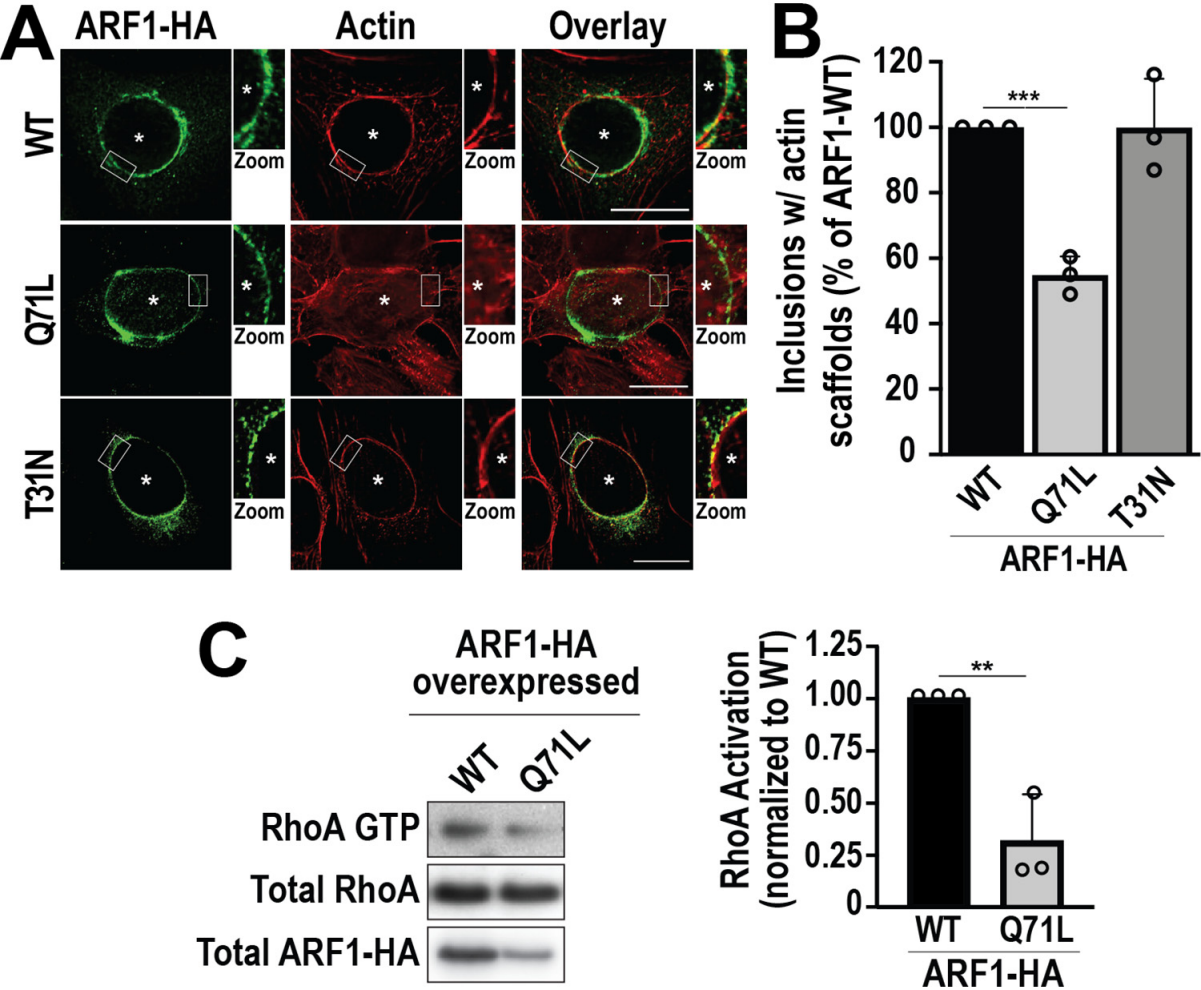
Activation of ARF1 impairs RhoA-GTP-dependent actin scaffold formation. (A) HeLa cells were infected with WT C. trachomatis L2 (MOI of 2) and transfected with ARF1_WT_-HA, ARF1_Q71L_-HA, or ARF1_T31N_-HA DNA at 4 hpi. Cells were fixed at 32 hpi and labeled with anti-HA antibody (green) and phalloidin (red). Asterisks denote inclusions. Scale bar, 20 μm. The white box indicates the magnified area shown to the right of the image (Zoom). (B) The graph depicts the average percentage of inclusions with actin scaffolds from 3 independent experiments ± the standard deviation. Data are normalized to cells transfected with ARF1_WT_-HA DNA. A minimum of 100 inclusions was counted for each condition from 3 independent experiments. ***, *P* < 0.001. (C) HeLa cells were infected with WT C. trachomatis L2 (MOI of 1) and transfected with ARF1_WT_-HA or ARF1_Q71L_-HA DNA at the time of infection. The levels of RhoA-GTP were determined as described in Fig. 1C. The graph depicts the average fold change of RhoA-GTP normalized to ARF_WT_-HA expressing cells from 3 independent experiments ± the standard deviation. **, *P* < 0.01.

The recruitment of RhoA to the inclusion begins around 24 hpi (Fig. S2). During this time, ARF1 is already activated, and PTM-MT scaffolds are forming. Since (i) the absence of RhoA concomitantly leads to the loss of actin scaffolds and enhances the interaction between ARF1-GTP and InaC (Fig. 4D) and (ii) ARF1 is not required for the formation of actin scaffolds (see Fig. S8 in the supplemental material), we hypothesized that the deactivation of ARF1 might be required for the subsequent downstream activation of RhoA and the formation of actin scaffolds. To test this hypothesis, we expressed the constitutively active ARF1_Q71L_-HA and dominant-negative ARF1_T31N_-HA mutants at 3 to 4 hpi with WT C. trachomatis L2 and assessed the formation of actin scaffolds at 32 hpi. Interestingly, the constitutive activation of ARF1 drastically reduces actin scaffold formation by 45% (Fig. 5A and B, ARF1_Q71L_-HA). This effect is specific to the active form of ARF1, as the expression of GDP-bound ARF1 does not influence actin scaffold formation (Fig. 5A and B, ARF1_T31N_-HA).

10.1128/mBio.02397-21.8 FIG S8 ARF1 is not required for actin scaffold formation during infection. (A to C) HeLa cells were transfected with nontargeting or hARF1 siRNA 48 h prior to infection with WT C. trachomatis L2 (MOI of 2). (A) At 32 hpi, cell lysates were analyzed by Western blotting to confirm knockdown of ARF1. HSP70 was used as a loading control. (B) Cells transfected with nontargeting or hARF1 siRNA 48 h prior to infection were fixed at 32 hpi and labeled with phalloidin (green) and anti-MOMP (blue) antibody. Scale bar, 20 μm. Asterisks denote inclusions. The white box indicates the magnified area shown to the right of the image (Zoom). (C) The graph denotes the average percentage of inclusions with actin scaffolds normalized to cells treated with the nontargeting siRNA from 3 independent experiments ± the standard deviation. A minimum of 100 inclusions was counted for each condition per experiment. Download FIG S8, PDF file, 10.9 MB. Copyright © 2021 Haines et al. 2021 Haines et al. https://creativecommons.org/licenses/by/4.0/ This content is distributed under the terms of the Creative Commons Attribution 4.0 International license.

Next, we determined whether the constitutive activation of ARF1 blocks the activation of RhoA. As shown in Fig. 5C, the activation of RhoA is inhibited by 68% ± 22% in cells expressing ARF1_Q71L_-HA compared to cells transfected with ARF1_WT_-HA. Collectively, these data demonstrate that cross talk between ARF1 and RhoA is critical for the coordination of PTM-MT and actin scaffolds during C. trachomatis infection.

**FIG 6:**
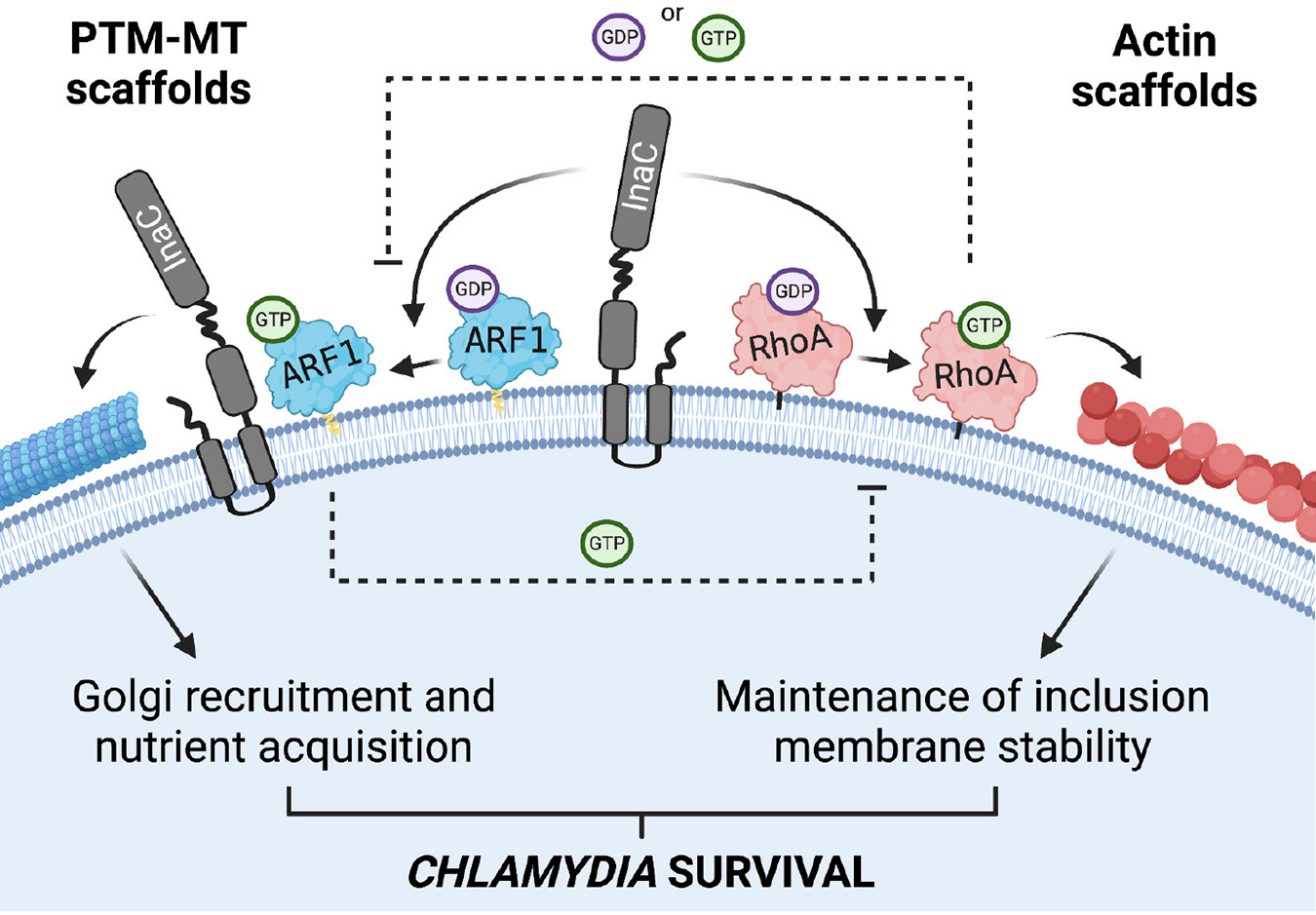
Model of cytoskeletal cross talk during Chlamydia infection. Chlamydia trachomatis expresses the inclusion membrane protein InaC on the surface of its inclusion facing the host cytosol. InaC is required to activate the host GTPases ARF1 and RhoA on the inclusion membrane and, consequently, for the formation of both PTM-MT and actin scaffolds, respectively. Both PTM-MT and actin scaffolds are essential for Chlamydia development and survival. ARF1-GTP is involved in the posttranslational modification of MT scaffolds around the inclusion, which supports Golgi recruitment and nutrient acquisition. ARF1 activation peaks early during infection. As RhoA begins to accumulate on the inclusion ∼24 hpi, its presence locally inhibits ARF1 activation and prevents the formation of additional PTM-MTs. Simultaneously, the presence of ARF1-GTP prevents the activation of RhoA and the formation of actin scaffolds early during infection. As ARF1 becomes deactivated and RhoA-GTP reaches its maximum, the formation of actin scaffolds begins. The continual presence of RhoA prevents PTM-MT scaffolds from unnecessarily forming late during infection. The accumulation of actin scaffolds around the inclusion ensures the maximal stability of the inclusion membrane. Figure created with BioRender.com.

Together, our data support a model in which Chlamydia uses a single effector to coordinate two small GTPases and regulate critical cytoskeletal pathways necessary for its survival (Fig. 6). Specifically, ARF1 is activated midcycle (∼16 hpi) in an InaC-dependent manner. At this time, RhoA is absent from the inclusion, leaving ARF1 activation unimpeded. As a result, ARF1-GTP actively drives the formation of PTM-MTs. In turn, PTM-MT scaffolds coordinate Golgi rearrangement around the inclusion, which contributes to inclusion growth (15). Around 24 hpi, RhoA starts to relocate to the inclusion membrane. The presence of RhoA blocks ARF1 activation and the generation of additional PTM-MTs. Simultaneously, ARF1-GTP inhibits actin scaffold formation by interfering with RhoA activation. As the infection progresses, the concentration of RhoA on the inclusion membrane increases, further inhibiting ARF1 activation. As ARF1 returns to its inactive GDP-bound state, it can no longer block RhoA activation. The InaC-dependent activation of RhoA fully engages, peaking at 32 hpi and maximizing actin polymerization around the inclusion. As a result, Chlamydia’s infectious niche, which has grown significantly due to the continuous supply of lipids from Golgi ministacks, is now fortified against premature lysis and ultimately provides a safe space for RBs to differentiate into infectious EBs and perpetuate the infectious cycle.

## DISCUSSION

Here, we have identified a virulence pathway in which the bacterial pathogen Chlamydia trachomatis uses a single chlamydial effector to integrate ARF1 and RhoA cross talk to precisely time the formation of PTM-MT and actin scaffolds (Fig. 6). Many other intracellular pathogens also target GTPases (29) and integrate GTPase cross talk into their virulence systems. An example is the effector protein EspG expressed by enteropathogenic E. coli OH157:H7, which disrupts ER-to-Golgi trafficking by linking ARF1-specific vesicular trafficking events to Rab1 inactivation (30, 31). EspG also blocks phagocytosis by interacting with ARF1 to prevent Rac1 activation and consequently the polymerization of actin at the plasma membrane (32). In contrast, Salmonella enterica co-opts an ARF6-ARF1-Rac1 axis to drive plasma membrane ruffling required for entry (33). The Chlamydia virulence pathway is most similar to that utilized by Salmonella since Chlamydia activates rather than inhibits the ARF1-RhoA network to promote successful infection. In this pathway, both RhoA and ARF1 elegantly fine-tune their activity by generating simultaneous feedback loops. The activation of ARF1 prevents the activation of RhoA, while the presence of RhoA downregulates ARF1 activation. While GTPases are well known to regulate each other (34–37), how RhoA and ARF1 influence the function of each other is unknown. Furthermore, whether this process requires other bacterial factors or is an inherent property of these GTPases and their respective effector proteins is under investigation.

Since GTPases are critical components of host signaling networks, manipulating their function or activity can have detrimental consequences for the host. In particular, dysregulation of GTPases involved in coordinating the host cytoskeleton has been linked to cancer hallmarks, such as uncontrolled cell proliferation, migration, and differentiation (38, 39). ARF1 and RhoA have been implicated in some aspects of cancer development, including tumor cell invasion and proliferation (40–45). Given that Chlamydia infection has been associated with an increased risk for developing cervical and ovarian cancers (46–48), it would be interesting to determine whether the activation of the ARF1-RhoA signaling axis contributes to the potentiation of cancer development in those with a history of chronic Chlamydia infection.

As an absolute intracellular bacterium, Chlamydia must ensure the integrity of its intracellular niche to establish a successful infection. As Chlamydia replicates inside the inclusion, this bacterial compartment matures and expands to eventually occupy most of the host cytoplasm (49). We have previously shown that InaC KO Chlamydia displays smaller inclusions and is replication deficient (15). These defects may be due to a combination of factors. (i) They may be a consequence of reduced PTM-MT scaffold formation, which impairs the recruitment of Golgi ministacks around the inclusion that play a role in lipid acquisition and inclusion development (50). (ii) Premature lysis could result in the release of Chlamydia into the cytoplasm before the cells have successfully differentiated from RBs into infectious EBs. We show here that InaC KO inclusions lyse significantly earlier than their WT counterparts (Fig. 3A). Since the infected cells then undergo more cell death (Fig. 3B) due to cytoplasmic Chlamydia, the bacterium reaches an impasse. While both possibilities likely contribute to the survival of Chlamydia, our data demonstrate that the chlamydial effector protein InaC is critical for the protection of Chlamydia's intracellular niche and successful infection by controlling two cytoskeletal networks.

InaC is required to activate both ARF1 and RhoA, which is tightly regulated during Chlamydia infection (Fig. 2) (15). This suggests that InaC may possess intrinsic GEF activity. However, we have shown that InaC does not exhibit GEF activity *in vitro* (15), indicating that other factors likely activate ARF1 and RhoA. How these GTPases are being inactivated and how this inactivation is coordinated are also unknown. Due to the significant number of ARF1 and RhoA GEFs and GTPase-activating proteins (GAPs) (51, 52), it is challenging to postulate what specific regulators may be involved. Nevertheless, GEFs containing GAP domains (52), or GAPs that have both ARF and RhoA GAP domains (53), may be critical for coupling the activities of ARF1 and RhoA.

Our data demonstrate that PTM-MT and actin scaffold formation occurs with distinct kinetics and that disruption of one of these pathways impacts the other. For example, we show that the constitutive activation of ARF1 blocks the formation of actin scaffolds (Fig. 5). Why can't actin scaffolds form in the presence of PTM-MT scaffolds? One possibility is steric hindrance. Since PTM-MTs woven around the inclusion possess enhanced stability and rigidity, the presence of these cytoskeletal elements might limit access to the inclusion membrane either for actin effectors to interact with chlamydial effectors or for the polymerization of actin itself. Additionally, PTM-MT and actin scaffolds may share critical components for their formation. We have already identified InaC as such a regulator; however, other host effectors may also be shared between these two pathways. For example, host formins control the generation of PTM-MTs and the polymerization of actin filaments (54). Thus, the activation of ARF1-dependent PTM-MTs may sequester critical components away from the actin pathway until ARF1 is inactivated, and these effectors can then participate in RhoA-dependent actin scaffold formation. Identification of these components will provide insight into how Chlamydia coordinates the specific kinetics of each of these cytoskeletal scaffolds.

While the activation of ARF1 blocks actin scaffold formation, the loss of RhoA results in the enhanced interaction between ARF1 and InaC and increased PTM-MT scaffold formation (Fig. 4). This phenotype can be reversed by the expression of RhoA in any of its nucleotide-bound states (Fig. 4; Fig. S7). The nucleotide independence of this complementation suggests that (i) the presence of RhoA on the inclusion is sufficient to downregulate ARF1 or (ii) RhoA may interact with effectors irrespective of its nucleotide-bound state to shut off ARF1. Several reports have documented that RhoA and other GTPases interact with other proteins in a nucleotide-independent manner to control their localization and activation (55–57). Thus, not only has Chlamydia co-opted two host GTPases to coordinate the formation of two cytoskeletal scaffolds, but it has also taken advantage of both nucleotide-dependent and -independent functions of these GTPases.

In summary, our findings have revealed a mechanism by which Chlamydia utilizes a single effector protein to control two cytoskeletal pathways. By employing GTPase cross talk, Chlamydia temporally regulates the assembly of PTM-MT and actin scaffolds around its inclusion to maintain its parasitic niche. Ultimately, identifying the molecular players participating in these pathways will uncover new targets to combat Chlamydia trachomatis infection.

## MATERIALS AND METHODS

### Cell culture and transfections.

HeLa cells (CCL-2; ATCC) and Nuclight Red HeLa cells (Essen Biosciences) were cultured as described previously in Dulbecco’s modified Eagle’s medium (DMEM) containing 10% fetal bovine serum (FBS), 10 μg/ml gentamicin, and nonessential amino acids (15). Cells were transfected with Continuum transfection reagent (Gemini Bioproducts) using 25 to 50 ng of DNA according to the manufacturer's instructions. For siRNA transfections, HeLa cells were transfected using 25 nM siRNA and DharmaFect 1 reagent (Horizon) according to the manufacturer’s instructions 48 h before infection. SmartPool ON-TARGETplus human ARF1 and nontargeting control siRNA were purchased from Dharmacon (15).

### Chlamydia strains.

Wild-type Chlamydia trachomatis serovar L2 (LGV 434/Bu) and InaC KO L2 were obtained from Ted Hackstadt (NIH, Rocky Mountain Laboratories) (15). C. trachomatis L2 was propagated and density gradient purified as described preiously (58, 59). The InaC KO and InaC-FLAG C. trachomatis L2 strains were generated previously (15, 60). InaC-FLAG-expressing InaC KO C. trachomatis L2 was produced by transforming InaC KO C. trachomatis L2 with InaC-FLAG pBomb4S-Tet encoding InaC-FLAG under the control of a tetracycline promoter as previously described (15).

### Recombinant DNA and cloning.

PCR and cloning were conducted using standard procedures. ARF1_WT_-HA and ARF1_Q71L_-HA were generated as previously described (15). ARF1_T31N_-HA was made by PCR amplification of ARF1_T31N_ (J. Keen, Thomas Jefferson University) and ligation into pcDNA3.1(+) containing a C-terminal HA tag. myc-RhoA_WT_ was amplified and ligated into pRK5 containing an N-terminal myc tag. myc-RhoA_G14V_ and myc-RhoA_T31N_ were generated using QuikChange PCR (Agilent) according to the manufacturer’s instructions. InaC-FLAG pBomb4S-Tet was generated by exchanging the green fluorescent protein (GFP) cassette in CT813-FLAG pBomb4-Tet (15) with a spectinomycin resistance cassette.

### Antibodies.

The following primary antibodies were used: anti-HA (chicken, A190-106A; Bethyl Laboratories), anti-actin (rabbit, A2066; Sigma), anti-myc (mouse, 9E10 clone; Juan Bonifacino, NIH), anti-myc (rabbit, 2278; Cell Signaling), anti-RhoA (mouse, ARH04; Cytoskeleton), anti-RhoA (mouse, SAB1400017; Sigma), anti-FLAG (mouse, F1804; Sigma), anti-FLAG (rabbit, 600-401-383; Rockland Immunochemical), anti-ARF1 (mouse, sc-53168; SCBT), anti-α-tubulin (mouse, T5168; Sigma), anti-acetylated α-tubulin (mouse, T6793; Sigma), anti-detyrosinated α-tubulin (rabbit, 48389; Abcam), ActiStain-488 (PHDG1; Cytoskeleton), Actistain-555 (PHDH1-A; Cytoskeleton), anti-heat shock protein 70 (HSP70) (chicken, SPC-178D; StressMarq), anti-InaC (mouse; T. Hackstadt), anti-IncA (rabbit; T. Hackstadt), and anti-MOMP (goat, 1621; ViroStat). The following secondary reagents were used: Hoechst dye (H1399) and goat and donkey anti-mouse, anti-goat, anti-rabbit, and anti-chicken (IgY) IgG Alexa Fluor 488-, 555-, or 647-conjugated secondary antibodies (Invitrogen). Donkey anti-chicken, anti-mouse, or anti-rabbit IgG and IgY horseradish peroxidase (HRP)-conjugated secondary antibodies were purchased from Invitrogen.

### Generation and characterization of the RhoA KO CRISPR/Cas9 cell line.

Guide RNAs targeting human RhoA was designed using the sequences available in GenBank and analyzed for their efficiency and off-target effects, using the CRISPOR (61) and the Broad Institute single guide RNA (sgRNA) designer (62) databases with default cutoffs. The optimal guide RNA was prepared by annealing primers FO1033 (5′-CAC CGG AAC TAT GTG GCA GAT ATC G-3′) and FO1034 (5′-AAA CCG ATA TCT GCC ACA TAG TTC C-3′). The guide RNA was then inserted into the BsbI site of the pSpCas9(BB)-2A-GFP (PX458) vector (S. Kim, Thomas Jefferson University) and confirmed by Sanger sequencing (Genewiz). HeLa cells were then transfected with pSpCas9(BB)-2A-GFP containing the guide RNA as well as the empty vector (no guide RNA) as described previously (63). Individual clones were isolated by limited dilution. Successful knockout of RhoA was confirmed by Western blotting. Cell division analysis using carboxyfluorescein succinimidyl ester (CFSE) labeling was conducted as described previously (63).

### Preparation of cell lysates for Western blot analysis.

Cells were washed with PBS and lysed with ice-cold lithium dodecyl sulfate (LDS)-PAGE sample buffer (Invitrogen) containing 250 U/μl benzonase (Accelagen), 1 μg/ml pepstatin A, 5 μg/ml leupeptin, 1 mM phenylmethylsulfonyl fluoride, 10 mM sodium fluoride, and 5.4 mM sodium orthovanadate for 10 min on ice. β-Mercaptoethanol was then added to a final concentration of 0.36 M at 95°C for 5 min, followed by centrifugation at 20,000 × *g* at 4°C for 10 min. Protein concentrations were determined using the Pierce 660-nm protein assay reagent containing ionic detergent compatibility reagent and read at 660 nm in a SpectraMax M2 plate reader.

### Western blotting.

Samples were separated on 4- to 12% bis-Tris SDS-PAGE gels (Invitrogen) and transferred to polyvinylidene difluoride membranes for 1 h at 90 V and 4°C in transfer buffer (25 mM Tris, 192 mM glycine, 10% methanol). Membranes were washed in TBS (25 mM Tris base, 150 mM NaCl [pH 7.5]) and then dried at room temperature for 1 h. Membranes were rehydrated with methanol and washed with TBS and TBST (TBS containing 0.1% Tween 20). Membranes were then blocked for 1 h at room temperature with blocking buffer (3% bovine serum albumin and 0.05% sodium azide in TBST). After blocking, membranes were incubated in primary antibody overnight at 4°C in blocking buffer. Membranes were washed with TBST before incubation with HRP-conjugated secondary antibody for 1 h at room temperature in 0.5% milk diluted in TBST. Membranes were then washed several times with TBST and TBS and revealed with SuperSignal West Dura extended duration substrate (Thermo Scientific). Membranes were imaged on a FluorChem R system (ProteinSimple). Band intensities were quantified using AlphaView software (ProteinSimple).

### Inclusion lysis analysis.

Cells were infected with WT or InaC KO C. trachomatis L2 at a multiplicity of infection (MOI) of 0.5 and fixed at the indicated times. When noted, WT C. trachomatis L2-infected cells were treated with either 0.5 μM latrunculin B or ethanol (vehicle control) in cell culture medium for 30 min at 37°C 5 h prior to fixation. Latrunculin B and ethanol were washed out with cell culture medium, and cells were fixed at the indicated times. For complementation of inclusion stability, HeLa cells were infected with WT or InaC-FLAG-expressing InaC KO C. trachomatis L2 at an MOI of 0.5 and fixed at 48 hpi. Inclusion integrity was determined by labeling inclusions with IncA and identifying discontinuity in the labeling of IncA on the inclusion membrane. An inclusion was considered broken or lysed when one or more substantial gaps in the incidence of IncA labeling were observed using wide-field fluorescence microscopy. Most of the broken inclusions were missing large pieces of the inclusion membrane. When inclusion integrity was less clear, the presence of Chlamydia in the cytosol was used to identify a broken inclusion.

### Immunofluorescence microscopy.

HeLa cells were fixed with either (i) 4% paraformaldehyde in PEMS buffer (80 mM PIPES, 5 mM EDTA, 2 mM MgCl_2_, 50 mM sucrose [pH 6.8]) for 15 min at room temperature or (ii) ice-cold methanol for 10 min at room temperature. Cells were then washed in IF-G buffer (25 mM HEPES, 150 mM NaCl, 900 nM CaCl_2_, 500 nM MgCl_2_, 100 mM glycine [pH 7.5]) for paraformaldehyde fixation or IF buffer (25 mM HEPES, 150 mM NaCl, 900 nM CaCl_2_, 500 nM MgCl_2_ [pH 7.5]) for methanol fixation. Permeabilization was performed with 0.2% Triton X-100 in IF buffer for 10 min, followed by washes with 0.1% Triton X-100 in IF buffer. Cells were then blocked for 1 h with either (i) goat serum (10% goat serum, 0.05% sodium azide, 0.1% Triton X-100 in IF buffer) or (ii) donkey serum (10% donkey serum, 0.05% sodium azide, 0.1% Triton X-100 in IF buffer) blocking buffer. After blocking, cells were treated with the primary antibodies diluted in the appropriate blocking buffer for 1 h at room temperature. Cells were washed with 0.1% Triton X-100 in IF buffer and subsequently incubated with Alexa Fluor-conjugated secondary antibodies and Hoechst, diluted in the appropriate blocking buffer for 1 h at room temperature. Cells were washed 0.1% Triton X-100 in IF buffer, followed by multiple washes with IF buffer, and mounted on coverslips with ProLong glass antifade mounting medium (Invitrogen). For inclusion lysis experiments, permeabilization, blocking, and subsequent washes were performed in 0.1% saponin instead of Triton X-100. A minimum of 100 cells per condition were observed and imaged with a 60× oil immersion lens on a Nikon TiE inverted fluorescence microscope and Elements software (Nikon). Images were processed using ImageJ (NIH).

### ARF1 and RhoA activation assays.

Cells were seeded at a density of 2.9 × 10^6^ cells per 150-mm tissue culture dish 24 h before infection with the indicated strains at an effective MOI of 1. Each plate of cells was lysed at 24 hpi (for ARF1) or 32 hpi (for RhoA) in 700 μl of 2× lysis buffer (50 mM Tris, 10 mM MgCl_2_, 0.5 M NaCl, 2% Igepal [pH 7.5]) containing protease inhibitor cocktail (K1007; APExBio) and immediately clarified by centrifugation at 20,000 × *g* for 1 min at 4°C. Lysates were rapidly frozen in liquid nitrogen and stored at −80°C. Isolation of ARF1-GTP was performed as described previously (15), using GST-GGA1 agarose and 800 μg of cell lysate diluted to 1× lysis buffer using distilled water. RhoA-GTP was isolated using a similar protocol, except the GST-GGA1 was replaced with the GST-Rhotekin RBD agarose beads (BK036; Cytoskeleton). The amount of active GTPase was determined by dividing the ARF1-GTP or RhoA-GTP signal from the pulldown with the ARF1 or RhoA signal from the cell lysate (total GTPase).

### Immunoprecipitation.

Prior to lysis, cells were fixed in 0.6% paraformaldehyde for 20 min at 4°C. Fixed cells were quenched with multiple washes in IF-G buffer. HeLa cells were lysed in ice-cold lysis buffer as described previously (15). Lysates were clarified by centrifugation, and equal amounts of protein were incubated overnight at 4°C with an anti-FLAG or anti-myc antibody immobilized on protein G Plus agarose beads. The beads were washed with lysis buffer and boiled at 95°C for 5 min in NuPAGE LDS sample buffer (Invitrogen). All samples were analyzed by Western blotting.

### Caspase 3/7 assay.

IncuCyte HeLa Nuclight Red cells (Essen Biosciences) were seeded into 96-well plates at a density of 7.5 × 10^3^ cells per well 24 h before infection with WT C. trachomatis L2 or InaC KO C. trachomatis L2 at an MOI of 1. The infection was synchronized by centrifugation at 1,000 × *g* for 1 h at room temperature. At 6 hpi, the IncuCyte caspase 3/7 green dye for apoptosis (Essen Biosciences) was added to each well at a final concentration of 5 μM. Wells were imaged every 2 h until 72 hpi using an IncuCyte Zoom imaging system (Essen Biosciences). The percentage of caspase 3/7-positive cells was calculated by dividing the number of caspase 3/7 puncta by the number of red nuclei.

### Statistical analysis.

A two-tailed Student's *t* test was employed when comparing the means from two independent groups. GraphPad Prism 9 was used for all statistical testing and data analysis. *P* values of <0.05 were considered statistically significant.

### Data availability.

The authors declare that all other relevant data supporting the findings of this study are included in the manuscript and its supplemental files or from the corresponding author upon request.
