# Supplementary figures and images for: Cross Talk between ARF1 and RhoA Coordinates the Formation of Cytoskeletal Scaffolds during Chlamydia Infection
Source: mBio. 2021 Dec 14;12(6):e02397-21. doi: 10.1128/mBio.02397-21 (PMC8669492; doi:10.1128/mBio.02397-21)

**A**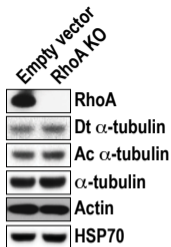**B**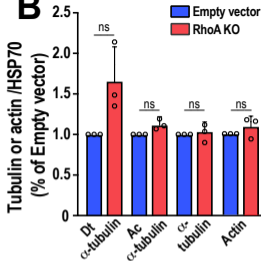**C**

RhoA CRISPR/Cas9  
KO Cell line

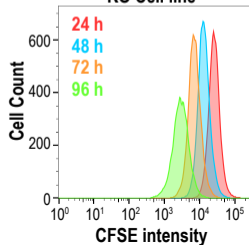**D**

Empty Cas9 vector  
control cell line

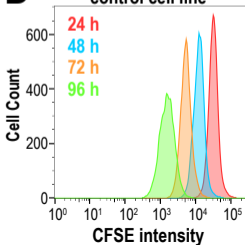

Supplement: FIG S1 [file mbio.02397-21-sf001.pdf]

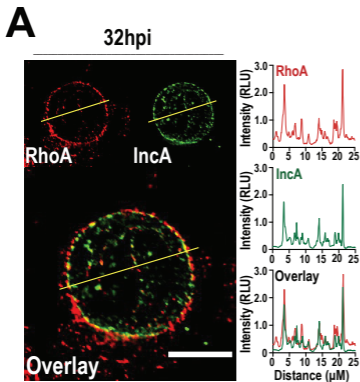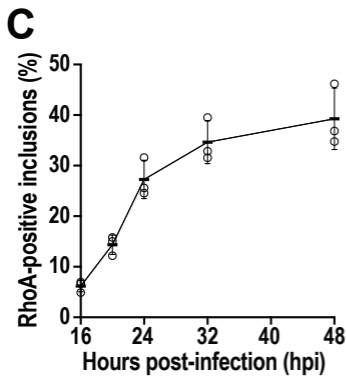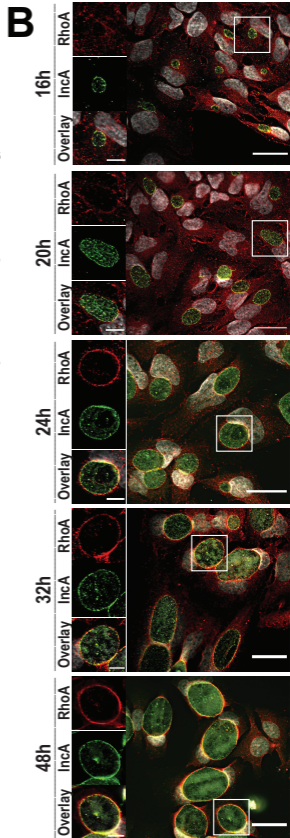

Supplement: FIG S2 [file mbio.02397-21-sf002.pdf]

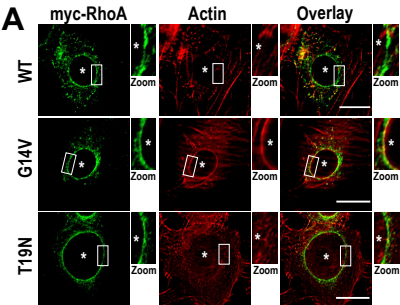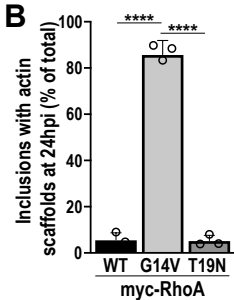

Supplement: FIG S3 [file mbio.02397-21-sf003.pdf]

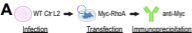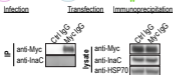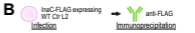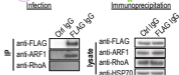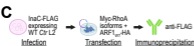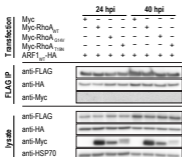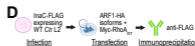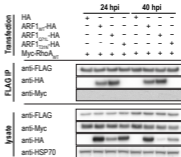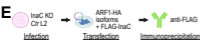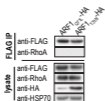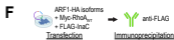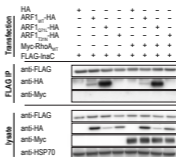

Supplement: FIG S4 [file mbio.02397-21-sf004.pdf]

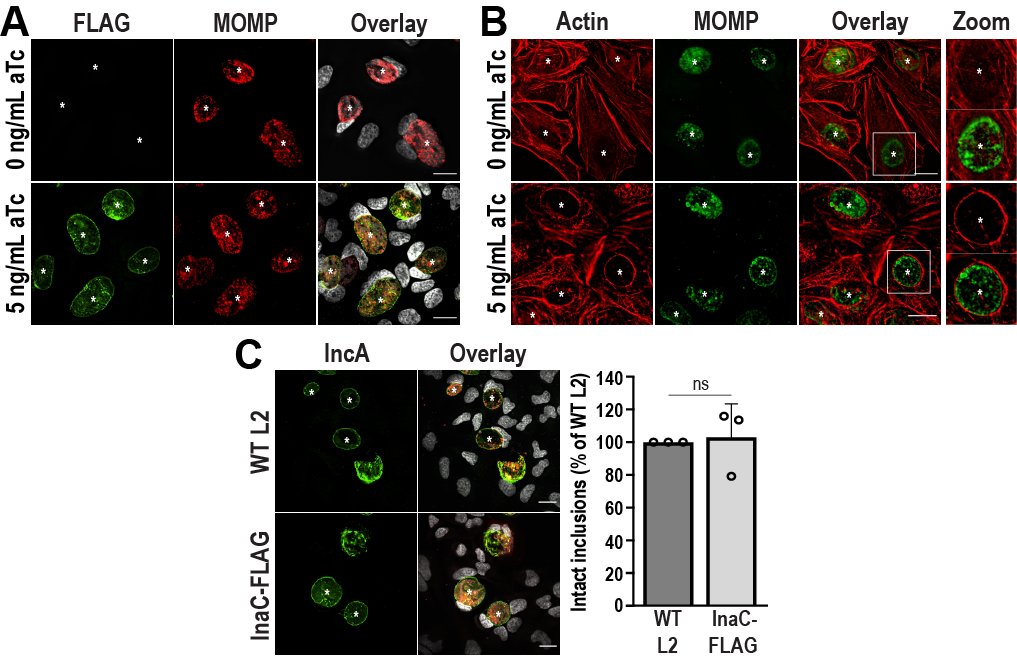

Supplement: FIG S5 [file mbio.02397-21-sf005.tif]

Not infected

L2-infected

8 16 24 32 40 48

8 16 24 32 40 48

hpi

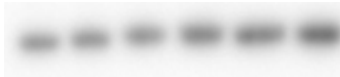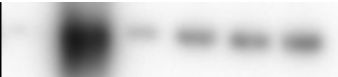

**ARF1 GTP**

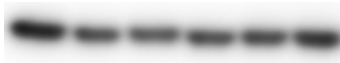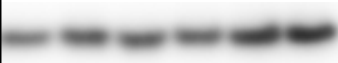

**Total ARF1**

Supplement: FIG S6 [file mbio.02397-21-sf006.pdf]

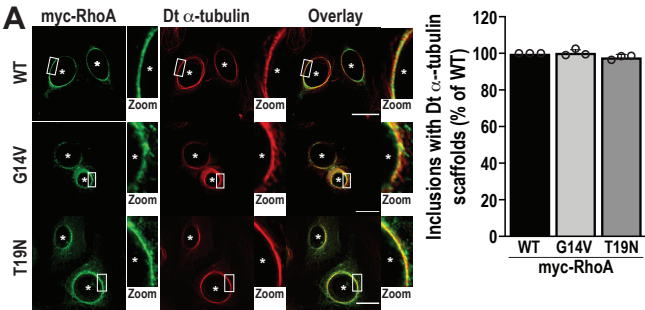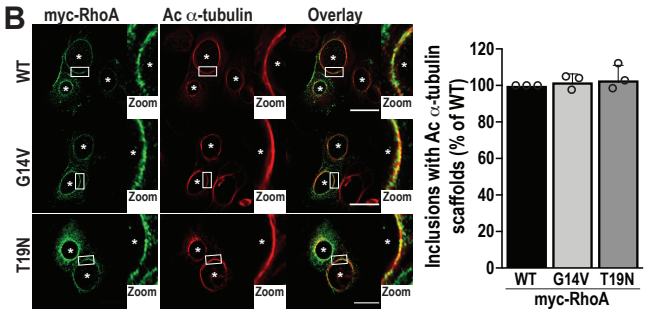

Supplement: FIG S7 [file mbio.02397-21-sf007.pdf]

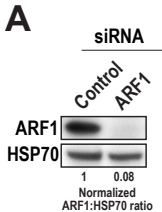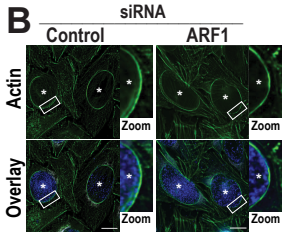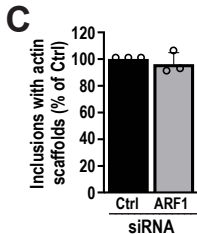

Supplement: FIG S8 [file mbio.02397-21-sf008.pdf]
